# Genome Projector: zoomable genome map with multiple views

**DOI:** 10.1186/1471-2105-10-31

**Published:** 2009-01-23

**Authors:** Kazuharu Arakawa, Satoshi Tamaki, Nobuaki Kono, Nobuhiro Kido, Keita Ikegami, Ryu Ogawa, Masaru Tomita

**Affiliations:** 1 Institute for Advanced Biosciences, Keio University, Fujisawa, 252-8520, Japan

## Abstract

**Background:**

Molecular biology data exist on diverse scales, from the level of molecules to -omics. At the same time, the data at each scale can be categorised into multiple layers, such as the genome, transcriptome, proteome, metabolome, and biochemical pathways. Due to the highly multi-layer and multi-dimensional nature of biological information, software interfaces for database browsing should provide an intuitive interface that allows for rapid migration across different views and scales. The Zoomable User Interface (ZUI) and tabbed browsing have proven successful for this purpose in other areas, especially to navigate the vast information in the World Wide Web.

**Results:**

This paper presents Genome Projector, a Web-based gateway for genomics information with a zoomable user interface using Google Maps API, equipped with four seamlessly accessible and searchable views: a circular genome map, a traditional genome map, a biochemical pathways map, and a DNA walk map. The Web application for 320 bacterial genomes is available at . All data and software including the source code, documentations, and development API are freely available under the GNU General Public License. Zoomable maps can be easily created from any image file using the development API, and an online data mapping service for Genome Projector is also available at our Web site.

**Conclusion:**

Genome Projector is an intuitive Web application for browsing genomics information, implemented with a zoomable user interface and tabbed browsing utilising Google Maps API and Asynchronous JavaScript and XML (AJAX) technology.

## Background

In molecular biology, it is important to look at reactions and behaviours of specific molecular components, both at the micro-level and at the macro-level, which we refer to as -omics. Biologists therefore must constantly traverse across micro-, meso-, and macro-levels of biological knowledge to gain insight into the workings of the cell. Moreover, our current understanding of cellular phenomena is also highly multi-layered, organised as assemblages of several -omic spaces such as the genome, transcriptome, proteome, metabolome, and biochemical pathways [1]. Each of these layers represents a projection of cellular anatomy containing unique sets of molecules and interactions and is often epitomised by distinct, high-throughput experimental technology for the comprehensive measurement of constituent entities. -Omic layers also provide a biological context for the visualisation and interpretation of data, and serve as a gateway to information stored in a database [2]. For example, the popular two-dimensional genome map viewer, Gbrowse, provides an entry point for browsing the model organism genome database, GMOD [3], and biochemical pathway databases such as KEGG [4], BioCyc [4], and Reactome [5] can be visually explored from illustrated pathway maps.

However, due to the availability of these public databases on the World Wide Web, a technical difficulty in representing the comprehensive set of the highly complex data in each of these -omic layers, within a single seamless graphic, has resulted in limitations in the user interface. The majority of current Web interfaces require several steps of user interactions to browse through the diverse scales, with delays between them for HTTP transactions. For example, to browse the biochemical pathway involved with glyceraldehyde-3-phosphate in KEGG, a user would start from an abstracted overview of the Metabolic Pathways map (map01100), click to proceed to the Carbohydrate Metabolism map (map01101), and then finally click to go to the Glycolysis/Gluconeogenesis map (map00010), which has enough resolution to show member enzymes and compounds. Subsequently, one would then have to switch between this map and an adjacent but separate map of the Pentose Phosphate Pathway (map00030). A similar user interaction is required for genome browsers to start from the chromosome illustrations and then focus in on a certain chromosomal locus so that the genes of interest are finally visible on the map.

The recent introduction of the Asynchronous JavaScript and XML (AJAX) and the so-called Web 2.0 paradigm allows for the realisation of the development of Web-based applications with a more intuitive user experience, and these developments overcome technical limitations [6]. With this new development paradigm, the Zoomable User Interface (ZUI) has proven to be effective to browse scalable data, especially with the success of Google Maps for multi-scale geographical information [7]. ZUI is also utilised for the visualisation of biological data [4,8-10], and the next versions of Reactome [11] and GBrowse [12] using AJAX ZUI are under development at the time of this writing.

Existing databases, however, are also typically equipped with only a single view mode and are thus specific to one layer of -omics, making comparative browsing among multiple layers difficult. For example, to see whether two adjacent enzymes in a biochemical pathway are coded closely together within a genome, one would have to access different databases implemented with incompatible interfaces. Therefore, to allow for intuitive browsing of multi-omic information for a given organism, it is desirable for a database browser to have ZUI, as well as seamless access from multiple view modes with a consistent user interface. Tabbed browsing for Web pages is a popular implementation for such a purpose.

In light of these requirements for an accessible user interface, this report introduces Genome Projector, a searchable genome browser with ZUI using Google Maps API and tabbed browsing for multiple -omic layers, in particular focusing on the chromosomal organisation of genetic elements in bacterial genomes. Genome Projector currently contains 4 views for 320 bacterial genomes: a circular genome map, a traditional genome map, a biochemical pathway map, and a DNA walk map.

## Implementation

Genome Projector is developed with G-language Genome Analysis Environment version 1.8.4 [13-15] with Ext-JS framework 1.0.1 for Web interfaces using the AJAX Web programming paradigm [16] and Google Maps API [17] for ZUI. Google Maps API was chosen for ZUI because of its high performance, ability to zoom with mouse scroll wheel operation, and popularity, so that users are already accustomed to the interface and therefore can navigate intuitively.

### Map generation and backend database

Large images of the genome maps (8192 × 8192 pixels) were generated using GD and SVG Perl modules from 320 circular bacterial genomes downloaded from the RefSeq FTP site [18], and images for the biochemical pathway map were obtained from the ExPASy server and merged [19]. These images were split into 256 × 256 pixel regions using the ImageMagick utility [20] to prepare them for the Google Maps API. Google Maps API loads images using Mercator projection coordinates, so the inter-conversions among this coordinate system, genomic position, and image pixel coordinates, as well as search queries, are processed by CGI scripts upon AJAX calls. Because the Web interface (View) is separated from the data source (Model), any backend database can be used with appropriate CGI handler (Controller). By default, Genome Projector uses tab-delimited flatfiles similar to General Feature Format (GFF), generated with Restauro-G bacterial genome re-annotation software [21]. Restauro-G adds annotations from the UniProt KnowledgeBase [22], NCBI COGs [23], Pfam [24], and PSORTdb [25] to the given genome flatfiles. EC numbers are used as primary identifiers instead of genomic positions for the biochemical pathway map.

## Results and discussion

**Figure 1:**
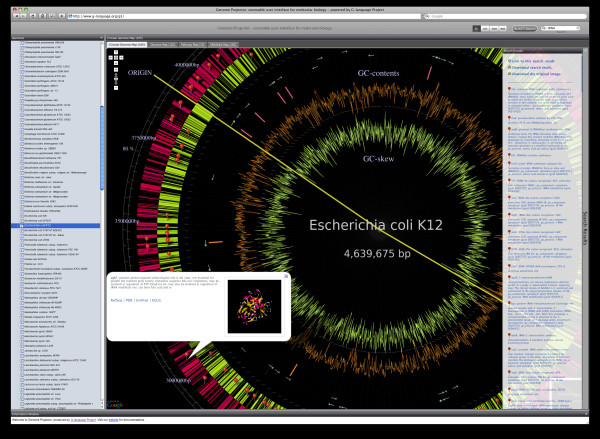
**Screenshot of Genome Projector**. This screenshot shows the circular genome map of *Escherichia coli *K12 searched with the keyword "tRNA" for all annotated genes and the detailed information window for the *ygfZ *gene. The leftmost pane shows the list of available genomes, the upper-right corner contains the search box, the rightmost pane shows the search results, and the main window contains the zoomable maps that can be readily switched using the tabs above.

Genome Projector is available from the project Web site [26] as a Web-based application, along with detailed documentation, a downloadable software package for installation, and a development Application Programming Interface (API). Figure 1 shows a screenshot of Genome Projector with the circular genome map of *Escherichia coli *K12. When a genome is selected from the leftmost pane, the zoomable map in the centre panel is immediately updated without page transition. Users can search through genome annotations from the search box located at the top right corner. Search results are listed in the rightmost panel and their corresponding locations within the map are shown with markers (red pins). Here, the search target can be limited to all genes, tRNAs, and rRNAs by selecting the corresponding switches located next to the search box. Keyword search goes through the entire annotation stored in the backend database, and regular expressions are allowed for experts. For example, "thr [A-Z]" searches for genes named *thrA*, *thrB*..., *thrZ*, and "(thr|rrn)" searches for genes containing either "thr" or "rrn" in their annotations. For the Pathway Map, enzymes can be searched by the compound names that the enzyme catalyzes. Alternatively, GenomeProjector can be searched using sequence similarity based on BLAST. Clicking on the "BLAST Search" button located next to the keyword search box opens up a new window where a user inputs a sequence of interest. Here the sequence can be raw sequence or single or multiple FASTA formatted entries of both nucleotide and amino acid. Type of the molecule (whether nucleotide or amino acid), corresponding program (blastn or blastp), and backend database (genome or proteins) are automatically interpreted. With sequence-based search, E-values are shown in the Search Result panel next to the gene names.

Clicking on the markers or objects in the map opens up an information balloon, which shows a summary of annotations, links to external databases including KEGG, PDB, UniProt, and NCBI, and a 3D protein structure when it is available from the Protein Data Bank (PDB) [27]. The main region showing the map can be used in the same way as Google Maps; for example, mouse click and drag moves the visible region, and mouse scroll wheel or double clicking allows zooming. Map type can be toggled using the four tabs (Circular Genome Map, Genome Map, Pathway Map, and DNA Walk Map) located on top of the map window, and search results are conserved throughout the different maps. Therefore, users can observe the genomic information from many -omic contexts to see, for example, how certain genes of interest are distributed relative to the replication origin and terminus, how they are co-located, and how they act together in the biochemical pathway. Search results and map type are also conserved upon selection of different genomes, both for keyword and sequence-based searches, which allows for a comparative study among various bacterial species. Search results can be downloaded in tab-delimited text format or as sharable URLs from the search result pane. Genome Map and Pathway Map also contain an overlay map that can be toggled with buttons located in the top-right corner of the maps. Full-size image of the maps can also be downloaded from the Search Results tab for further local manipulation.

### Circular Genome Map

**Figure 2:**
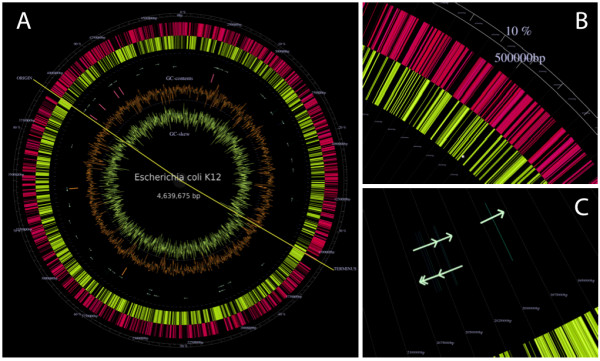
**Circular Genome Map**. Circular Genome Map view represents the genome in circular form, a visualisation approach typical for circular bacterial chromosomes and plasmids. (A) Map of the *Escherichia coli *K12 genome. From the outer ring inwards are genes on the direct strand (red), genes on the complementary strand (yellow), tRNAs (green arrows), rRNAs (pink or orange stripes depending on the strand), GC content (brown lines), GC skew (yellow lines). Replication origin and terminus predicted from the cumulative GC skew shift points at single base pair resolution are also labelled, with a yellow line cutting through the genome, segregating the two replichores. This view is useful to see the chromosomal organisation of genes, especially those related to replication. (B) A close up of the region around 500,000 bp. Genes on the direct strand are represented with pink lines and those on the complementary strand are marked in yellow. (C) Close up around the aspartyl-tRNA operon. The strand of tRNA is represented by the direction of the arrowhead. Because tRNAs are very short (typically around 75 bp), their exact positions are also marked with perpendicular stripes.

The Circular Genome Map represents the genome in circular form, a visualisation approach typical for circular bacterial chromosomes and plasmids and useful for seeing the chromosomal organisation of genes, especially in relation to replication (Figure 2A). The outermost red and yellow rings represent the positions of genes. The outer red ring corresponds to the direct strand of genome flatfile annotation, and the inner yellow ring corresponds to the complementary strand (Figure 2B). Each stripe represents a single gene, with the thickness corresponding to the length of the gene. Coordinates of the gene positions are labelled both inside and outside of these two rings. Circular bacterial genomes have a single pair of replication origin and terminus, which is marked by a long yellow line running through the rings, dividing the genome into two segments. Moving clockwise from the replication origin to the terminus, the outer red ring is the leading strand and the inner yellow ring is the lagging strand.

Moving inwards, tRNAs are represented by arrows, directed in the orientation as stated in the genome flatfile: clock-wise when direct, anti-clockwise when complementary (Figure 2C). Because tRNAs are relatively short compared with coding genes (about 75 bp in length compared with 1 kbp of coding genes), the length of the arrows is much longer than the actual length of tRNAs. Therefore, the exact locations of tRNAs are also marked with perpendicular stripes, similar to the representation of genes in the outer rings. The stripe is blue for the direct strand and green for the complementary strand. rRNAs are represented by pink and orange stripes, depending on the strand. rRNAs tend to strongly prefer the leading strand, and in some genomes, many rRNAs are located close to the replication origin. The copy numbers of tRNAs and rRNAs have been suggested to correlate with the growth rate of bacteria [28] and are indicative of the locations of other genes selected by the replication machinery, such as essential genes and operons [29-31].

The two innermost rings represent the GC content (brown lines) and GC skew (yellow lines) graphs, calculated with 2000 bp windows sliding 1000 bp each. GC skew is the excess of C over G in given regions, formulated as (C-G)/(C+G) [32,33]. In bacterial genomes, replicational selection prefers guanine over cytosine in leading strands; therefore, negative GC skew value is typically observed in leading strands and positive skew in lagging strands. In fact, GC skew is often utilised to define the positions of the replication origin and terminus in bacterial genomes. In Genome Projector, a thin green ring that runs through the GC skew ring indicates the zero position, and values inward of this ring are negative, and those outward are positive. Although this green ring is not visible in lower zoom depth, note that the distance between the rings of GC-contents and GC-skew is larger for negative valued region corresponding to the leading strand. The replication origin and terminus are predicted from the cumulative GC skew shift points at single base pair resolution [34].

### Genome Map

**Figure 3:**
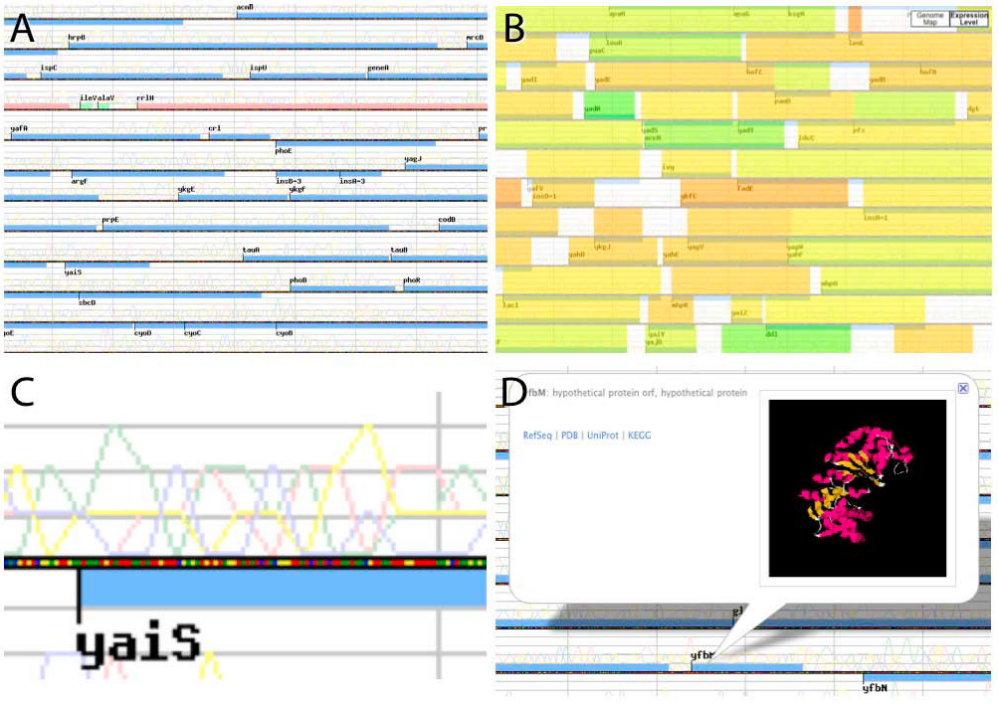
**Genome Map**. Genome Map view represents the genome in traditional genome browser layout, where genomic features (i.e., genes) are displayed with boxes around a linear chromosome laid out continuously in stacked rows. (A) CDS are marked with blue stripes, tRNA with green stripes, and rRNA with red stripes. (B) Information can be overlaid on top of the genome map as semi-transparent layers, which can be toggled with a switch located at the upper right corner. In Genome Projector, predicted gene expression levels are calculated with the Codon Adaptation Index and visualised with a colour spectrum ranging from red to green, corresponding to a CAI of 0 to 1. (C) Local nucleotide content is shown both as graphs (A: red, T: green, G: yellow, C: Blue) and as coloured pixels. (D) All objects (CDS, tRNA, rRNA) are clickable to display more information without search result markers.

The Genome Map represents the genome in a traditional genome browser layout, where genomic features (i.e., genes) are displayed with boxes around linear chromosomes laid out continuously in stacked rows. Three features are displayed in the genome map view: CDS with blue stripes, rRNA with red stripes, and tRNA with green stripes. Gene names are labelled at the left side of the stripes, corresponding to the 5'-end of genes located in the direct strand, located above the coloured dotted lines, and to the 3'-end of genes in the complementary strand, located below the line (Figure 3A). This view is useful to see the lengths of gene and their neighbours, overlapping or forming operons (polycistrons) that are transcribed together as a single mRNA. tRNA genes are often transcribed together as operons; therefore, clustering of green stripes is observed in many bacterial genomes. Likewise, operons usually share identical prefixes in gene names and have similar biological functions. Because bacterial genomes are highly compact, large percentages of genes overlap [35,36]. In many bacterial genomes, genes are also preferentially located on the leading strand; therefore, genomes with a highly skewed architecture in this respect have a continuous strand preference that reverses halfway through the genome.

At the maximum zoom level, the line representing the chromosome (the horizontal line in the middle in the following image) is composed of coloured pixels showing the actual nucleotide sequences (Figure 3C). In most bacterial genomes, each pixel represents a single base, but in larger genomes, each pixel may represent the most frequent base among several bases. Above the line is a graph of averaged nucleotide content visualised like chromatograms. Here, A is shown in red, T in green, G in yellow, and C in blue.

Similar to the hybrid satellite map in Google Maps, semi-transparent layers can be overlaid on the Genome Map, which can be toggled with buttons located in the upper-right corner (Figure 3B). In Genome Projector, the overlay for the Genome Map shows the predicted gene expression levels calculated using the Codon Adaptation Index (CAI) [37]. CAI measures the relative adaptiveness of the synonymous codon usage bias of a gene towards that of highly expressed genes, usually using ribosomal protein-coding genes as a reference set. CAI ranges from 0 to 1, which is represented by a colour spectrum ranging from red to green, respectively.

### Pathway Map

**Figure 4:**
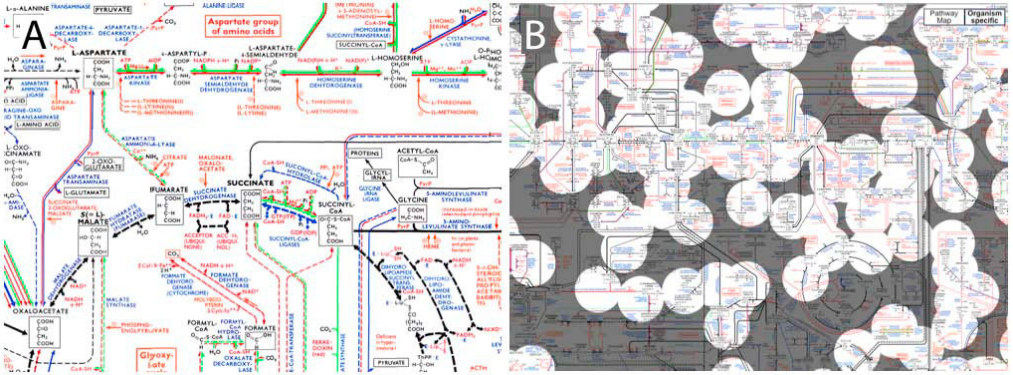
**Pathway Map**. Pathway Map view in Genome Projector is based on the Roche Biochemical Pathway wall chart, which provides a biochemical context of the reactome and metabolome. (A) Here, enzymes are shown in blue letters, coenzymes in red, and other compounds in black. Lines in black represent general pathways, red represents unicellular organisms and fungi, blue represents animals, and green represents higher plants. Orange lines show regulatory pathways, accompanied by + or - signs to show activation or downregulation. Key compounds have chemical structures shown in boxes bound by black borders. Every enzyme (in blue text) is clickable to show more detailed information. (B) The overlay layer highlights the organism specific enzymes and pathways by greying out the enzymes that are not annotated in the given organism.

The Pathway Map in Genome Projector is based on the Roche Biochemical Pathway wall chart available from the ExPASy proteomics server [38]. This view provides a biochemical context of the reactome and metabolome (Figure 4A). Here, enzymes are shown in blue letters, coenzymes in red, and other compounds in black. Lines in black represent general pathways, red for unicellular organisms and fungi, blue is for animals, and green is for higher plants. Orange lines show regulatory pathways, accompanied by + or - signs to show activation or down regulation. Key compounds have chemical structures shown in boxes bound by black borders. Every enzyme (in blue text) is clickable to show more detailed information.

The Roche Biochemical Pathway wall chart is extremely comprehensive compared with other existing pathway databases, because it displays the reactions, enzymes, metabolites, chemical structures of compounds, activations and inhibitions, and reversibility of reactions, while displaying the entire pathways collectively within one view. Although the majority of the existing pathway databases intentionally hide some of this information or subdivide the pathways, the Roche Biochemical Pathway wall chart allows system biologists to gain a comprehensive understanding of cellular components at a glance. Because of the large size of the map and the details within, the Roche Biochemical Pathway wall chart is an ideal platform to be viewed using ZUI.

The Roche Biochemical Pathway wall chart shows the collection of all biochemically known molecules, so the enzymes and reactions depicted here are not necessarily present in all organisms. Using the semi-transparent overlay layer that can be toggled with the buttons located in the top-right corner, Genome Projector highlights only the enzymes that are present in the specified genome and greys out the rest (Figure 4B). Here, the presence of enzymes in a genome is identified by matching the EC number between the KEGG and ExPASy ENZYME databases.

### DNA Walk Map

DNA Walk is a vectorial representation of DNA sequences transformed into a planar trajectory [39]. Two pairs of complementary nucleotides (A-T, G-C) are suitable for a two dimensional vectorisation, so the DNA sequence is visualised by drawing the trajectory of nucleotides moved upwards for A, downwards for T, to the right for G, and to the left for C. DNA Walk is therefore the integrated representation of GC skew and AT skew and, conversely, GC skew can be considered the projection of DNA Walk in the GC vector. The origin of DNA Walk (i.e., position 1 in a genome flatfile) is marked by a cross-section of grey axes, and nucleotides change colour from red to green as the position of the given nucleotide progresses within the sequence.

**Figure 5:**
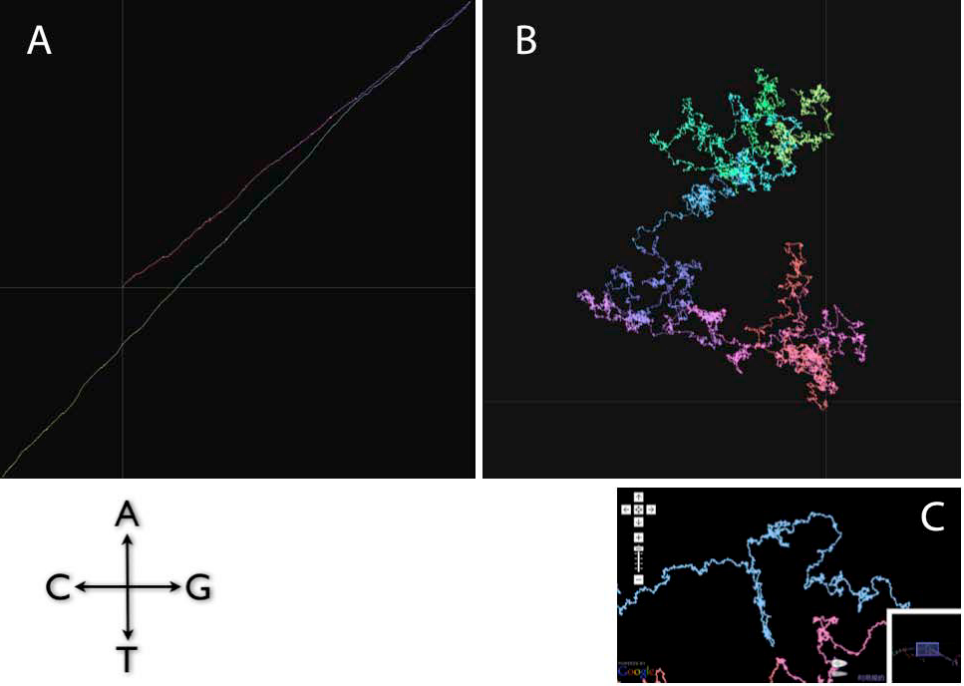
**DNA Walk Map**. DNA Walk is a vectorial representation of DNA sequences transformed into a planar trajectory, visualised by drawing the trajectory of nucleotides moved upwards for A, downwards for T, to the right for G, and to the left for C. (A) DNA Walk of *Clostridium perfringens*, which exhibits strong AT/GC skew and therefore produces an extremely linear V-shaped graph, whose vertices correspond to the replication origin and terminus. (B) DNA Walk of *Gloeobacter violaceus*, where no GC skew is observable. Large regions of asymmetric nucleotide compositions resulting in large hairpin-like structures can be observed. (C) Close-up of region around the *yfg *operon in *Escherichia coli*, where a large hairpin-like structure about 50 kilobases long can be observed.

DNA Walk reveals patterns in genomic sequences. Clustering of repeats, palindromes, horizontally transferred genes, telomeres, and GC skew can be easily spotted using this visualisation approach [40]. Figure 5A and 5B shows a highly selected example, a genome of *Clostridium perfringens *that shows extremely biased AT/GC skew resulting in a linear V-shaped visualisation, where the two converging lines correspond to the two replichores, and that of *Gloeobacter violaceus*, where no linear region is visibile and therefore no GC skew is observable. The linear segment corresponds to a region with continuously biased nucleotide content, and in bacterial species with circular chromosomes that contain only one finite origin of replication, a genome should be divided into two linear segments in the DNA Walk representation. In most genomes, a characteristic large hairpin-like structure can be observed at local regions of asymmetric nucleotide composition, in lengths of many kilobases (Figure 5C).

### Development API and data mapping service

The entire software system of Genome Projector is available as free software under the GNU General Public License for installation in custom Web servers, and the system can be used with other types of maps, backend databases, and genomes. Detailed documentation about system installation, use of custom databases and Perl programming API for the creation of four views available in Genome Projector with other genomes and for the creation of zoomable maps using Google Maps API from any image file, is available at the Web site [26].

To allow for quick mapping of experimental data onto Genome Projector using the semi-transparent overlay layer, we provide at our Web site a mapping service where users can upload their own data to be visualised on two of the Genome Projector maps, Genome Map and Pathway Map [41]. The required data format is basically a CSV (comma-separated vector) format with 3 columns (2 required and 1 optional), where each line represents an entry that contains the location or name of the entry, colour, and size (optional), similar to other pathway mapping services provided by the G-language Project [9,42]. Entry name can be specified by locus tags, gene names, EC numbers, or genomic positions.

### AJAX ZUI and tabbed browsing

Many genome browsers are currently available, including the aforementioned Gbrowse, UCSC Genome Browser, and Ensembl. As a genome browser, the functionality of Genome Projector is rather limited compared to these software tools, in terms of the amount of information contained, semantic zooming, and customizability. On the other hand, these software tools require page transition between the different zoom levels, and although that is not a problem in terms of functionality, the user experience of Genome Projector should provide a proof-of-concept of AJAX ZUI approach. Moreover, Genome Projector was designed not as just a genome browser, but as a browsing gateway for omics information, including genome, transcriptome, and biochemical pathways, which requires comparative perspective between multiple species and multiple viewpoints. To this end, Genome Projector provides quick and intuitive means to switch between the organisms and views with tabbed browsing user interface, so that certain gene of interest can be analyzed from different biological context. Coupling of ZUI and tabbed browsing is a unique feature provided by Genome Projector. It is also worth noting that although ZUI is available in local software such as Apollo [43] and with SVG-based web application BioViz [44], Genome Projector is readily usable without installation or browser plug-ins.

## Limitations

Genome Projector is currently limited to bacterial species with circular chromosomes due to a focus on the effects of genome organisation relative to replicational selection. Although the Circular Genome Map may only be applicable to these bacterial species, the overall interface implemented using AJAX and the visualisation concept utilising ZUI coupled with tabbed browsing should in principle be applicable to a variety of biological information. However, to extend the ZUI to eukaryotic genomes, which are 1000-fold larger than those of bacteria, the maximum zoom level should be extended 5 more levels ($\sqrt[{4}]{1000}$ roughly equals 5), which will require significantly larger computational time and disk space, and semantic zooming is preferable at such a depth. Moreover, since the Roche Biochemical Pathway wall chart lacks many plant specific pathways, use of other pathway database may be considered for the Pathway Map for the application of Genome Projector to plant genomes; nevertheless, ZUI approach itself should be applicable for these species.

Semantic zooming is a visualisation technique that changes the representation method depending on the magnification or zoom levels, mostly by altering the degree of abstraction at each level [45,46]. Most existing genome browsers, such as the UCSC genome browser [47], Ensembl [48], and Gbrowse, as well as the aforementioned pathway databases, take advantage of semantic zooming to present large amounts of information while allowing the users to browse in multiple scales. Because the current version of Genome Projector does not utilise semantic zooming, each of the maps contains less information compared with the above tools and databases developed for their specific purposes. However, semantic zooming can also be utilised with ZUI, and the Google Maps API supports the use of different abstractions at each zoom level. Enhancement of Genome Projector with semantic zooming and its application to eukaryotic genomes will be a focus of our future work.

## Conclusion

This paper reports the development of a Web-based application named Genome Projector, a searchable genome browser with ZUI using Google Maps API and tabbed browsing for multiple -omic layers, especially focusing on the chromosomal organisation of genetic elements in bacterial genomes. Genome Projector currently contains 4 views for 320 bacterial genomes: circular genome map, traditional genome map, biochemical pathway map, and DNA walk map. Genome Projector is useful as a gateway to multi-scale and multi-layered -omic information.

## Availability and requirements

**Project name**: Genome Projector

**Project home page**: 

**Operating system(s)**: Platform independent (Web application)

**Programming language**: Perl and Javascript

**Other requirements**: none

**License**: Web application is freely accessible for all users. Development API is available under GNU General Public License version 2.

**Any restrictions to use by non-academics**: none

## Abbreviations

AJAX: Asynchronous Javascript and XML; API: Application Programming Interface; DS: Coding Sequence; CGI: Common Gateway Interface; ZUI: Zoomable User Interface.

## Authors' contributions

KA conceived the system, developed the Web application framework and development API, and drafted the manuscript. ST developed the biochemical pathway view and backend database, NKo designed the circular genome map view and created the Web site, NKi developed the circular genome map view, KI developed the DNA walk view, and RO developed the genome map overlay. MT supervised the project. All authors read and approved the final manuscript.

## References

[B1] Toyoda T, Mochizuki Y, Player K, Heida N, Kobayashi N, Sakaki Y (2007). OmicBrowse: a browser of multidimensional omics annotations. Bioinformatics.

[B2] Francke C, Siezen RJ, Teusink B (2005). Reconstructing the metabolic network of a bacterium from its genome. Trends Microbiol.

[B3] Stein LD, Mungall C, Shu S, Caudy M, Mangone M, Day A, Nickerson E, Stajich JE, Harris TW, Arva A (2002). The generic genome browser: a building block for a model organism system database. Genome Res.

[B4] Kanehisa M, Araki M, Goto S, Hattori M, Hirakawa M, Itoh M, Katayama T, Kawashima S, Okuda S, Tokimatsu T (2008). KEGG for linking genomes to life and the environment. Nucleic Acids Res.

[B5] Matthews L, Gopinath G, Gillespie M, Caudy M, Croft D, de Bono B, Garapati P, Hemish J, Hermjakob H, Jassal B (2008). Reactome knowledgebase of human biological pathways and processes. Nucleic Acids Res.

[B6] Zhang Z, Cheung KH, Townsend JP (2008). Bringing Web 2.0 to bioinformatics. Brief Bioinform.

[B7] Google Maps. http://maps.google.com/.

[B8] Berger SI, Iyengar R, Ma'ayan A (2007). AVIS: AJAX Viewer of Interactive Signaling Networks. Bioinformatics.

[B9] Kono N, Arakawa K, Tomita M (2006). MEGU: pathway mapping web-service based on KEGG and SVG. In Silico Biol.

[B10] Okuda S, Yamada T, Hamajima M, Itoh M, Katayama T, Bork P, Goto S, Kanehisa M (2008). KEGG Atlas mapping for global analysis of metabolic pathways. Nucleic Acids Res.

[B11] beta-version of new Reactome interface.

[B12] GBrowse 2.0. http://gmod.org/wiki/GBrowse.

[B13] Arakawa K, Mori K, Ikeda K, Matsuzaki T, Kobayashi Y, Tomita M (2003). G-language Genome Analysis Environment: a workbench for nucleotide sequence data mining. Bioinformatics.

[B14] Arakawa K, Suzuki H, Tomita M (2008). Computational Genome Analysis Using The G-language System. Genes, Genomes and Genomics.

[B15] Arakawa K, Tomita M (2006). G-language System as a platform for large-scale analysis of high-throughput omics data. Journal of Pesticide Science.

[B16] Ext-JS framework. http://extjs.com/.

[B17] Google Maps API. http://code.google.com/apis/maps/.

[B18] Pruitt KD, Tatusova T, Klimke W, Maglott DR (2008). NCBI Reference Sequences: current status, policy and new initiatives. Nucleic Acids Res.

[B19] Schneider M, Tognolli M, Bairoch A (2004). The Swiss-Prot protein knowledgebase and ExPASy: providing the plant community with high quality proteomic data and tools. Plant Physiol Biochem.

[B20] ImageMagick. http://www.imagemagick.org/.

[B21] Tamaki S, Arakawa K, Kono N, Tomita M (2007). Restauro-G: a rapid genome re-annotation system for comparative genomics. Genomics Proteomics Bioinformatics.

[B22] UniProtConsortium (2008). The universal protein resource (UniProt). Nucleic Acids Res.

[B23] Tatusov RL, Fedorova ND, Jackson JD, Jacobs AR, Kiryutin B, Koonin EV, Krylov DM, Mazumder R, Mekhedov SL, Nikolskaya AN (2003). The COG database: an updated version includes eukaryotes. BMC Bioinformatics.

[B24] Bateman A, Birney E, Cerruti L, Durbin R, Etwiller L, Eddy SR, Griffiths-Jones S, Howe KL, Marshall M, Sonnhammer EL (2002). The Pfam protein families database. Nucleic Acids Res.

[B25] Gardy JL, Laird MR, Chen F, Rey S, Walsh CJ, Ester M, Brinkman FS (2005). PSORTb v.2.0: expanded prediction of bacterial protein subcellular localization and insights gained from comparative proteome analysis. Bioinformatics.

[B26] Genome Projector. http://www.g-language.org/GenomeProjector/.

[B27] Henrick K, Feng Z, Bluhm WF, Dimitropoulos D, Doreleijers JF, Dutta S, Flippen-Anderson JL, Ionides J, Kamada C, Krissinel E (2008). Remediation of the protein data bank archive. Nucleic Acids Res.

[B28] Sharp PM, Bailes E, Grocock RJ, Peden JF, Sockett RE (2005). Variation in the strength of selected codon usage bias among bacteria. Nucleic Acids Res.

[B29] Omont N, Kepes F (2004). Transcription/replication collisions cause bacterial transcription units to be longer on the leading strand of replication. Bioinformatics.

[B30] Price MN, Alm EJ, Arkin AP (2005). Interruptions in gene expression drive highly expressed operons to the leading strand of DNA replication. Nucleic Acids Res.

[B31] Rocha EP, Danchin A (2003). Essentiality, not expressiveness, drives gene-strand bias in bacteria. Nat Genet.

[B32] Lobry JR (1996). Asymmetric substitution patterns in the two DNA strands of bacteria. Mol Biol Evol.

[B33] Lobry JR, Louarn JM (2003). Polarisation of prokaryotic chromosomes. Curr Opin Microbiol.

[B34] Touchon M, Rocha EP (2008). From GC skews to wavelets: a gentle guide to the analysis of compositional asymmetries in genomic data. Biochimie.

[B35] Fukuda Y, Washio T, Tomita M (1999). Comparative study of overlapping genes in the genomes of Mycoplasma genitalium and Mycoplasma pneumoniae. Nucleic Acids Res.

[B36] Yachie N, Arakawa K, Tomita M (2006). On the interplay of gene positioning and the role of rho-independent terminators in Escherichia coli. FEBS Lett.

[B37] Sharp PM, Li WH (1987). The codon Adaptation Index – a measure of directional synonymous codon usage bias, and its potential applications. Nucleic Acids Res.

[B38] Gasteiger E, Gattiker A, Hoogland C, Ivanyi I, Appel RD, Bairoch A (2003). ExPASy: The proteomics server for in-depth protein knowledge and analysis. Nucleic Acids Res.

[B39] Peng CK, Buldyrev SV, Goldberger AL, Havlin S, Sciortino F, Simons M, Stanley HE (1992). Long-range correlations in nucleotide sequences. Nature.

[B40] Larionov S, Loskutov A, Ryadchenko E (2008). Chromosome evolution with naked eye: palindromic context of the life origin. Chaos.

[B41] Genome Projector data mapping service. http://www.g-language.org/g3/mapping/.

[B42] Arakawa K, Kono N, Yamada Y, Mori H, Tomita M (2005). KEGG-based pathway visualization tool for complex omics data. In Silico Biol.

[B43] Lewis SE, Searle SM, Harris N, Gibson M, Lyer V, Richter J, Wiel C, Bayraktaroglir L, Birney E, Crosby MA (2002). Apollo: a sequence annotation editor. Genome Biol.

[B44] BioViz. http://www.svgopen.org/2002/papers/lewis_et_al__bioviz_genome_viewer/.

[B45] Hu Z, Mellor J, Wu J, Kanehisa M, Stuart JM, DeLisi C (2007). Towards zoomable multidimensional maps of the cell. Nat Biotechnol.

[B46] Loraine AE, Helt GA (2002). Visualizing the genome: techniques for presenting human genome data and annotations. BMC Bioinformatics.

[B47] Kuhn RM, Karolchik D, Zweig AS, Wang T, Smith KE, Rosenbloom KR, Rhead B, Raney BJ, Pohl A, Pheasant M (2009). The UCSC Genome Browser Database: update 2009. Nucleic Acids Res.

[B48] Flicek P, Aken BL, Beal K, Ballester B, Caccamo M, Chen Y, Clarke L, Coates G, Cunningham F, Cutts T (2008). Ensembl 2008. Nucleic Acids Res.

